# The complete chloroplast genome of *Crataegus hupehensis* Sarg. (Rosaceae), a medicinal and edible plant in China

**DOI:** 10.1080/23802359.2020.1866464

**Published:** 2021-02-05

**Authors:** Guanglong Hu, Shuqi Zheng, Qinghua Pan, Ningguang Dong

**Affiliations:** Beijing Academy of Forestry and Pomology Sciences, Beijing Academy of Agriculture and Forestry Sciences, Beijing, P.R. China

**Keywords:** *Crataegus hupehensis*, chloroplast genome sequence, phylogenetic analysis

## Abstract

*Crataegus hupehensis* Sarg. is well-known for its medicinal and nutritive value. In this study, the complete chloroplast genome sequence of *C. hupehensis* was determined by using Illumina high-throughput sequencing approach. The complete chloroplast genome is 159,766 bp with 36.6% GC content. It contained a pair of inverted repeat regions of 26,385 bp, a large single-copy region of 87,852 bp, and a small single-copy region of 19,144 bp. It contained 112 distinct genes, including 78 protein-coding genes, 4 ribosomal RNA genes, and 30 transfer RNA genes. Phylogenetic analysis based on chloroplast genomes indicated that *C. hupehensis*is was closely related to *C. kansuensis* and *C. marshallii* in the subfamily Maloideae. This complete chloroplast genome will provide valuable insight into evolution, molecular breeding, and phylogenetic analysis of *Crataegus* species.

*Crataegus hupehensis* Sarg. (family: Rosaceae) is a medicinal and edible plant belonging to *Crataegus* species which are widely distributed in the temperate regions of the northern hemisphere in Europe, Asia, and North America (Phipps et al. 1990; Du et al. 2019). The genus *Crataegus* L., commonly known as hawthorn, are one of the most economically important plant groups in China, owing to their nutrient-rich fruit and significant medicinal values (Xu et al. 2016). Hawthorns contain bioactive components, such as flavonoids, phenols and oligomeric procyanidins, that are widely used in traditional Chinese medicine (Rigelsky and Sweet 2002; Dahmer and Scott 2010).

China is the main center of *Crataegus* cultivation, and the place of origin of both cultivated and some wild *Crataegus* species (Guo and Jiao 1995). A total of 18 species and six varieties of *Crataegus* are widely distributed across China (Zhao and Feng 1996). Among these taxa, *C. hupehensis*, *C. pinnatifida* var. *major*, *C. bretschneideri*, and *C. scabrifolia* are cultivated (Du et al. 2019; Ma et al. 2019). However, the interspecies relations and origins, and evolution of the four cultivated *Crataegus* species remain unknown. Furthermore, genomic resources of *Crataegus* species are limited. Therefore, we reported the complete chloroplast genome of *C. hupehensis* based on Illumina sequencing data (GenBank accession number MW201730), which would be helpful for molecular breeding and phylogenetic analysis.

A single individual of *C. hupehensis* was collected from the Hawthorn Germplasm Repository of Beijing Academy of Forestry and Pomology Sciences (39°97′N, 116°23′E) in Beijing, China. The voucher specimen (accession number: BJLGY-2020-SZ001) was deposited at the Herbarium of Beijing Academy of Forestry and Pomology Sciences (BAFPS-H, http://www.lgs.baafs.net.cn/, Yuanyong Qi, bjlgsbgs@126.com). DNA extraction was performed according to a modified CTAB protocol (Li et al. 2013) and paired-end libraries were prepared with the NEBNext Ultra DNA Library Prep Kit. High-throughput sequencing was carried out using the HiSeq Xten PE150 System (Illumina, San Diego, CA, USA) with150bp pair-end reads. In all, 1.17 G raw reads were obtained, and after the quality-trimmed using the software CLC Genomics Workbench v7.5 (CLC bio, Aarhus, Denmark), 0.97 G qualified reads were assembled using SPAdes 3.6.1 (Kmer = 95) (Bankevich et al. 2012) to contigs. The contigs of chloroplast genome were selected with the BLAST program (Altschul et al. 1990), taking the closely related species *C.marshallii* (MK920293) as a reference, and the selected contigs were assembled using Sequencher 4.10 (https://www.genecodes.com/) software tools. Annotation was performed using the Plann (Huang and Cronk 2015), then a physical map of the chloroplast genome generated by Genome Vx (Conant and Wolfe 2008).

The size of *C. hupehensis* chloroplast genome was 159,766 bp with 36.6% GC content. It contained a large single-copy (LSC) region of 87,852 bp, a small single-copy (SSC) region of 19,144 bp, and two inverted repeat (IR) regions of 26,385 bp. The chloroplast DNA of *C. hupehensis* comprised a total of 112 unique genes, including 78 protein-coding genes, 30 tRNA genes, and 4 rRNA genes. In these genes, 18 genes were duplicated in the IR regions, 15 genes harbored a single intron, and 2 (*ycf3, clpP*) contained double introns.

To clarify the phylogenetic position of *C. hupehensis*, total 31 complete chloroplast genomes were obtained from Genbank and the sister group Rosoideae was taken as an outgroup. All chloroplast genome sequences were aligned using MAFFT (Katoh et al. 2019) and phylogenetic analysis was conducted based on maximum-likelihood (ML) analyses using IQ-TREE (1.6.12) with 1000 bootstrap replicates (Nguyen et al. 2015). The phylogenetic analysis showed that *C. hupehensis* was closely related to *C. kansuensis* and *C. marshallii* in the subfamily Maloideae (Figure 1). This complete chloroplast genome can be used for future studies on genetic engineering, population and phylogeny of family Rosaceae.

**Figure 1.**
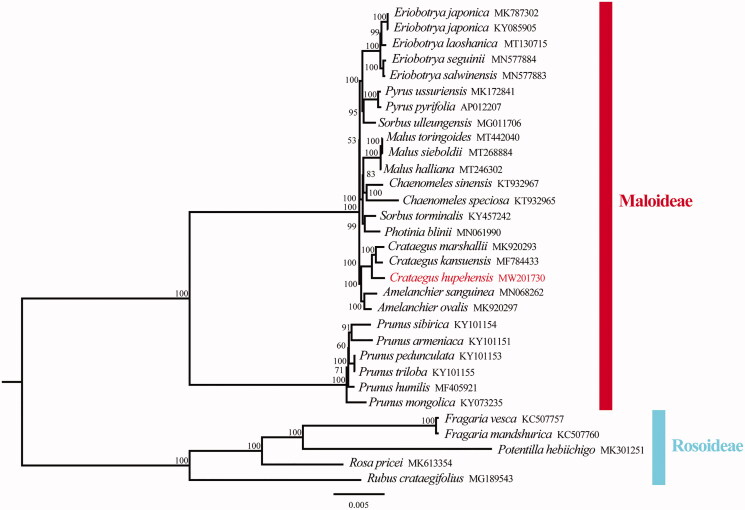
Phylogenetic tree reconstruction of 31 taxa using maximum likelihood (ML) methods based on the chloroplast genome sequences. ML bootstrap support value presented at each node.

## Data Availability

The genome sequence data that support the findings of this study are openly available in GenBank of NCBI at (https://www.ncbi.nlm.nih.gov/) under the accession no. MW201730. The associated BioProject, SRA, and Bio-Sample numbers are PRJN A660005, SUB8681476, and SAMN16998579 respectively.
